# The Importance of Predicting Bowel Necrosis in Acute Mesenteric Ischemia: Narrative Review

**DOI:** 10.3390/diagnostics16020332

**Published:** 2026-01-20

**Authors:** Caterina Giannessi, Diletta Cozzi, Ludovica Scalzone, Francesca Treballi, Matilde Anichini, Barbara Sessa, Anna Ferrarelli, Ginevra Danti, Vittorio Miele

**Affiliations:** 1 Department of Radiology, Careggi University Hospital, Largo Brambilla 3, 50134 Florence, Italy; 2 Department of Radiology, San Filippo Neri Hospital, Via Martinotti 20, 00135 Rome, Italy; 3 Department of Radiology, Great Metropolitan Hospital “Bianchi-Melacrino-Morelli”, Via Giuseppe Melacrino 21, 89124 Reggio Calabria, Italy; 4 Department of Experimental and Clinical Biomedical Sciences “Mario Serio”, University of Florence, 50121 Florence, Italy

**Keywords:** acute mesenteric ischemia, computed tomography, bowel necrosis, AAMI, AMVI, NOMI

## Abstract

Acute mesenteric ischemia (AMI) is a clinical condition caused by vascular insufficiency, resulting in intestinal damage. Is often underestimated, if not driven by clinical suspicion, due to the non-specific clinical symptoms (usually represented by acute abdominal pain) and the absence of reliable markers, which results in a poor prognosis and high mortality. We can identify three main forms of AMI: arterial, venous, and non-occlusive. Arterial AMI is the most frequent form, caused by occlusion of the superior mesenteric artery or one of its branches. Venous AMI is the least frequent, caused by thrombosis of the superior mesenteric vein or its branches. Non-occlusive AMI is due to a state of hypovolemia, which is frequent in patients who have undergone surgery. Given the difficulty of diagnosis based on the clinic alone, the radiologist plays a central role in identifying radiological signs of intestinal ischemia and in avoiding misdiagnosis. The radiologist’s role is mainly to identify factors predictive of necrosis, which allow us to stratify patients and direct them towards the proper management. The aim of this review is to provide indications for an adequate CT protocol, including an unenhanced phase, an arterial phase, and a venous phase, as well as to underline the features to investigate in the different forms of AMI, in order to increase the diagnostic capacity in this challenging disease.

## 1. Introduction

**Figure 1:**
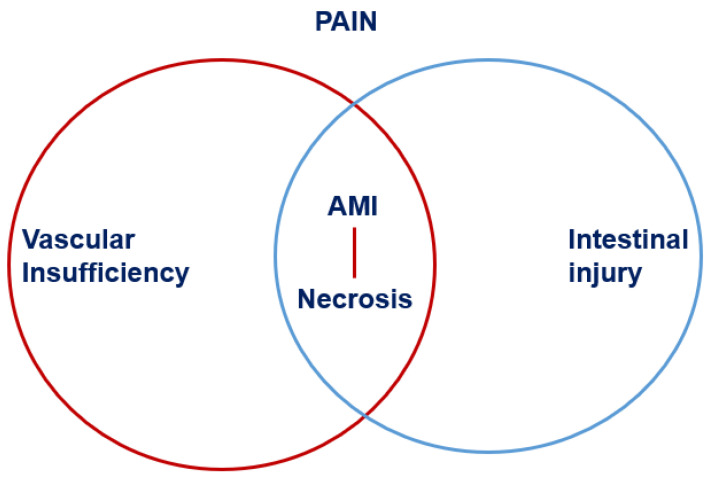
Definition of AMI.

Acute mesenteric ischemia is a life-threatening cause of acute abdominal pain, caused by a sudden decrease in blood supply to the gastrointestinal tract, resulting in cell damage, intestinal necrosis, and, if left untreated, death. AMI is defined as an intestinal injury caused by vascular insufficiency, as we see in Figure 1 [1].

Three main arteries are responsible for gastrointestinal blood supply: the celiac artery, the superior mesenteric artery (SMA), and the inferior mesenteric artery (IMA), with all of them originating from the ventral surface of the abdominal aorta [2]. These vessels are connected via anastomotic arches; therefore, the bowel can withstand a decreased flow of up to 75% for 12 h without suffering any damage [3].

The overall incidence varies around the world and is underestimated due to the diagnostic challenge that AMI represents: a recent observational multicenter study that involved 32 centers around the world (AMESI study) reported an incidence of AMI of 0.038% in hospitalized patients, with a mortality of 50% in patients with a confirmed diagnosis of AMI [4]. The incidence increases exponentially with age [5].

It is a condition with poor prognosis and elevated mortality: literature reports a more than 90% mortality rate without proper treatment in the late 1970s [6], while remaining unfortunately high even in recent reviews and meta-analyses, about 50–60% [7,8]. Therefore, despite its poor prognosis, research is very limited due to its low incidence [9]: it is indeed an uncommon cause of abdominal pain, yet a very common cause of emergency bowel resection.

In AMI, we can identify occlusive and non-occlusive causes and recognize different forms [10]:

- Acute Arterial Mesenteric Ischemia (AAMI): the most frequent, it accounts for approximately 60–85% of all causes of AMI [2,11]. It is caused by occlusion of the SMA or one of its branches due to embolic or thrombotic processes.
- Acute Venous Mesenteric Ischemia (AVMI): the least frequent due to venous thrombosis of the SMV or in one of its confluent branches [11].
- Non-occlusive Mesenteric Ischemia (NOMI): 5–15%, due to non-occlusive causes, essentially resulting in hypovolemia, absolute (e.g., hemorrhagic shock) or relative (septic shock, cardiogenic shock, major surgery, certain vasoactive substances, etc.) [11].

These are, therefore, low-flow states that lead to splanchnic vasoconstriction and acute intestinal circulatory insufficiency; if prolonged, it may lead to intestinal necrosis.

Distinguishing the various entities is crucial because they have different pathogenesis and different prognoses, whereas previous studies have analyzed patients by mixing up the various forms, thereby creating a bias [6,12].

The main diagnostic challenge in AMI is that the clinic is extremely non-specific: most patients present with severe abdominal pain with acute onset, requiring morphine. We may have other non-specific abdominal symptoms, but usually, on physical examination, the patient may only present with a tense abdomen. Unlike other types of ischemia, such as myocardial ischemia, we do not have specific or reliable laboratory tests or markers to guide us toward a diagnosis [13]. Indeed, several studies have focused on the predictive value of lactates: high lactate levels are detected in only 32–50% of patients with AMI, so a normal lactate value is not reassuring [14,15]. A recent study focused on three promising biomarkers, citrulline, I-FABP, and D-lactate, and showed that they are neither sensitive nor specific for AMI [13]. Moreover, Mihaileanu et al. pointed out that although these biomarkers related to vascular damage, inflammatory response, and tissue damage may have potential diagnostic and prognostic value, the different outcomes highlighted by the different studies prove that a standardized methodology and further research are needed [16].

Several studies underlined how AMI was underdiagnosed if not led by clinical suspicion [17]. Anglaret et al. investigated how clinical suspicion of AMI could influence CT accuracy, demonstrating that there were no differences in accuracy according to clinical suspicion, whereas AMI was not suspected in approximately 60% of the cases later confirmed on CT scans. It was proven that the negative impact of not having a clinical suspect was overcome by using an appropriate CT protocol [18].

According to the most recent literature, the only clinical factors that can lead to an early diagnosis are acute abdominal pain with rapid onset and/or requiring morphine, which should prompt a multiphasic CT scan with arterial and venous phase images [19].

Hence, AMI is indeed a diagnostic challenge. As there are no strong clinical or biological indicators, the condition of AMI is not often suggested by emergency department physicians, which reflects negatively on the adoption of a correct CT protocol. Garzelli et al. argue that the radiologist should consider the diagnosis of AMI in the presence of any sudden, acute abdominal pain [20]. CT is crucial in the diagnosis of AMI, and the radiologist plays a pivotal role in the diagnosis [21]. The two main factors that can lead to a diagnostic delay in AMI are normal plasma lactate values and unenhanced CT-scan [14].

A high radiologic expertise is needed, both to catch radiological signs predictive of necrosis and to avoid misdiagnosis due to the numerous conditions mimicking AMI and possible pitfalls [14,22,23], as abdominal CT scans are often performed in patients with non-specific acute abdominal pain.

## 2. CT Scan Protocol

For optimal CT imaging, patients should be positioned supine (lying on their back), and the scan should cover the area from the liver dome to the pubic symphysis. Multiplanar reformations (MPR) should be routinely performed to enhance the visualization of anatomical structures in multiple planes. A scan without contrast medium is strongly recommended because it is useful for assessing any hyperdense portions of the intestinal wall on unenhanced examination. In addition, according to Chuong et al., it increases sensibility and inter-observer agreement [24]. However, unenhanced scanning should always be combined with contrast medium scanning because CT scanning without contrast medium delays diagnosis and greatly increases mortality from AMI [25]. A contrast medium is essential for the visualization of splanchnic vessels and for enhancing intestinal loops. At least 2 mL/kg of contrast medium with a concentration of 350 mg/mL must be injected; the optimal injection rate is 4 mL/s for proper visualization of the arteries [26]. The administration of oral contrast medium is not recommended, as it increases CT duration, can cause beam hardening artifacts, and hinders the proper analysis of the parietal enhancement by decreasing the contrast between the wall and lumen [26,27]. The optimal protocol involves an arterial acquisition, which should be done with bolus-tracking or bolus-triggered technique with an enhancement threshold of 150 UH. The use of acquisition with a fixed delay should be discouraged because of the risk of missing the optimal arterial peak in patients with hemodynamic changes. Optimal arterial enhancement is useful for assessing the presence of arterial occlusion and determining whether there is a proximal or distal obstruction. Acquisition in the venous phase is recommended after 50 s compared to the arterial phase [28]. According to Nakhaei et al., the split bolus technique reduces the required contrast dose and results in fewer images to interpret compared to standard multiphase CTA. In their approach, they administered two boluses of contrast: the first (100–150 mL) for portal vein opacification, followed by a second bolus of 50 mL for the arterial phase, given 22–33 s later [29]. Dual-energy CT (DECT) with iodine maps enhances the assessment of tissue enhancement and vascularization compared to conventional single-energy CT. Iodine maps directly display contrast material enhancement without interference from background tissue density, improving diagnostic accuracy. In a study by Tommaso D’Angelo et al., DECT iodine maps significantly outperformed conventional CT for diagnosing non-occlusive mesenteric ischemia (NOMI), offering a more accurate evaluation of tissue perfusion and vascularization [30]. Moreover, some studies, such as the one conducted by Ota et al., show that the use of photon-counting detector CT enables better visualization of abdominal arteries, even the more peripheral ones [31].

## 3. Arterial Occlusive or Stenotic Mesenteric Ischemia (AAMI)

The most common type of arterial involvement in acute mesenteric ischemia (AMI) is the occlusion of the SMA, responsible for 60–85% of cases [11]. The occlusion is due to one of two main causes: calcified atherosclerotic thrombosis, which stems from a subacute process of plaque remodeling often seen in patients with multi-arterial disease, or a recent clot caused by an embolus from the heart or aorta. The exact prevalence of either phenomenon is difficult to quantify, as many studies have inconsistent data. According to the updated guidelines of the World Society of Emergency Surgery, the prevalence of mesenteric acute embolism has been reduced in recent years and amounts to approximately 25% of cases. In contrast, cases of mesenteric artery thrombosis have increased to account for about 40% of the causes of mesenteric ischemia [21]. A rare cause of AAMI is isolated acute spontaneous dissection of the mesenteric artery (<5% of all causes) [12].

Recent studies [32] show that identifying the exact location of the SMA occlusion is of the utmost importance, as this influences the therapeutic approach. The choice between open or endovascular surgery, and in the case of endovascular treatment, the choice between thrombectomy and in situ thrombolysis, depends on the location of the SMA occlusion [33]. Retrospective studies conducted show that in most of the reports, there is no precise description of the location of the occlusive lesions of the SMA, but it is referred to as ‘proximal’ or ‘distal’ [28].

A recent proposal by Tual et al. [34] suggested an anatomical segmentation of the SMA using computed tomography angiography (CTA) to help standardize the reporting of occlusive lesions in AMI. The SMA was divided into three segments: the proximal SMA, which extends from the ostium to the origin of the inferior pancreaticoduodenal artery; the middle SMA, from the origin of the inferior pancreaticoduodenal artery to the origin of the ileocolic artery; and the distal SMA, which includes the vascular territory beyond the ileocolic artery. Additionally, a detailed description of the involvement of the jejunal and colic arteries was included.

In atherosclerotic thrombosis, the SMA is affected by diffuse plaques, which can be either calcified or non-calcified, and these are most prominent around the ostium of the artery: at this level, stenosis is most frequently found [35]. Other arteries are often diseased, and stenosis of the coeliac trunk in association with SMA is common. In patients with atherosclerosis of the SMA, downstream vessels are usually opacified by collateral circulators, which ensure adequate base flow to the bowel [36]. AMI occurs predominantly because of a thrombotic process on the atherosclerotic plaque, leading to vessel occlusion. However, especially in patients with multivessel disease, a small reduction in flow may be sufficient to cause AMI, even without complete occlusion. Stenosis greater than 90% of the SMA or more than 70% stenosis of both the celiac trunk and the SMA are considered high-risk factors for AMI [36].

**Figure 2:**
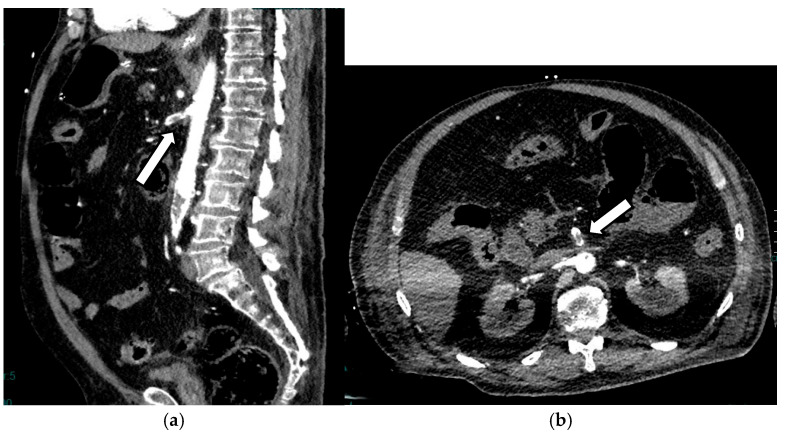
AAMI in a 78-year-old man: sagittal (**a**) and axial (**b**) contrast-enhanced CT scans show thrombotic occlusion at the emergence of the SMA (arrows).

In embolic AMI, patients are typically younger than those with atherosclerotic AMI, and the onset of the condition is often more abrupt. These individuals usually have fewer collateral vessels. Occlusion can affect any segment of the SMA, S1, S2, or S3, and is characterized by a sudden termination of contrast flow and a filling defect in the distal SMA [35]. Detecting the thrombus in the distal branches can be challenging, and a careful evaluation of the smaller collateral vessels of the SMA to aid in diagnosis is suggested. In most patients with atrial fibrillation, these emboli originate from the heart, particularly from the auricle of the left atrium [37]. Synchronous emboli may be present; in most cases, they are localized to the lower limbs [35]. The density of thrombus in the SMA may serve as an independent risk factor for transmural wall necrosis in patients with AMI due to SMA thromboembolism, with denser clots associated with a higher risk [38] (Figure 2).

### 3.1. Bowel Involvement in AAMI

Concerning bowel involvement, we can say that ‘time is gut.’ AMI is a dynamic process; in the first phase, we have ischemic damage to the intestine, which then evolves into transmural necrosis. Therefore, AMI is not a synonym for infarction: ischemia is only a step before the onset of necrosis and infarction, and mortality is drastically reduced if the patient is treated quickly (before 12 h from the onset of symptoms, we see mortality is 16% and then increases rapidly) [6].

As radiologists, we have not only a diagnostic role but also a prognostic role: in addition to assessing the association of vascular insufficiency and ischemic intestinal damage and thus diagnosing AMI, we must also look for the presence or absence of necrosis. The identification of necrosis significantly changes the therapeutic approach as it requires surgical exploration.

Table 1 shows the long list of features that are predictors of ischemic bowel necrosis, the radiologist’s role being to emphasize the importance of the main ones [39]. According to the recent meta-analysis by Zheng et al., thinning of the intestinal wall, decreased or absent intestinal wall enhancement, intestinal dilatation, intestinal pneumatosis, porto-mesenteric venous gas, and occlusive arterial AMI are the main risk factors for transmural intestinal necrosis [40]. Lactates were confirmed as unreliable markers of intestinal necrosis, although high lactate levels were strongly correlated with reduced pH and reduced bicarbonates as signs of shock, predictably correlating with poor prognosis [41].

High creatinine levels also correlated, while inflammatory markers did not prove reliable [41].

#### 3.1.1. Decreased/Absent Wall Enhancement

**Figure 3:**
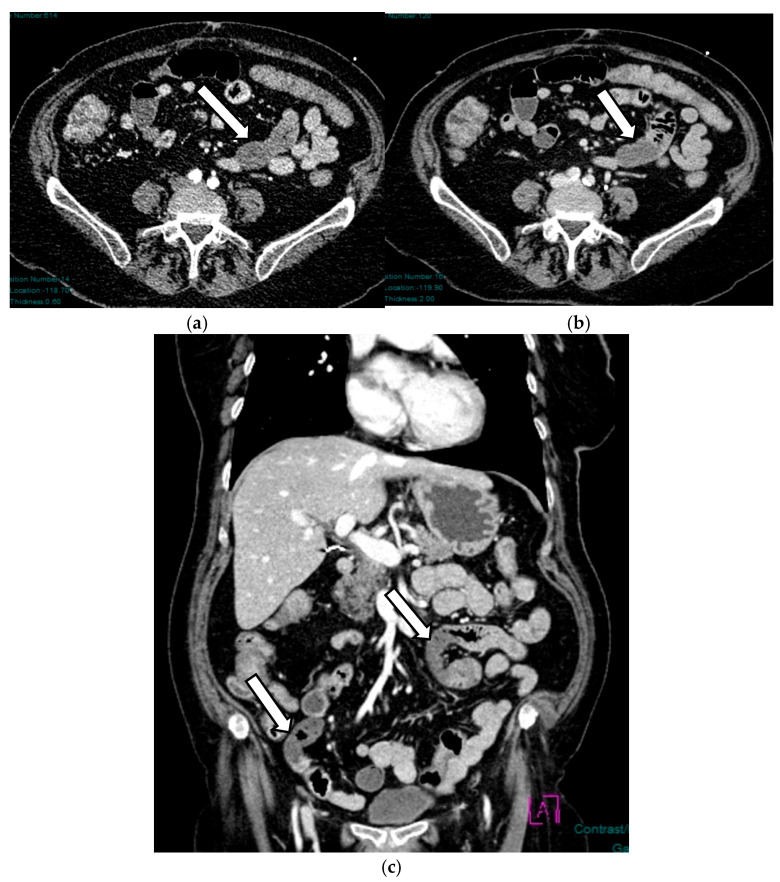
AAMI in a 64-year-old woman: axial (**a**,**b**) and coronal (**c**) post-contrast CT scans show some ileal loops with absent wall enhancement in the meso-hypogastric region, especially if compared to the adjacent loops (arrows).

In the early phase, the ischemic injury of the bowel in the early phase is mainly represented on imaging by decreased bowel wall enhancement, which typically reflects reduced perfusion to the intestines. We must evaluate wall enhancement and always compare non-contrast loops with normal wall enhancement loops. It has been seen that inter-reader agreement in identifying reduced wall enhancement is low [28] (Figure 3).

To improve hypoperfused loops evaluation, we must compare post-contrast CT with pre-contrast CT. Numerous studies prove that the comparison improves confidence and inter-reader agreement [24,42,43,44]. Reduced wall enhancement indicates intestinal ischemia with a specificity ranging from 97% in the study by Kirkpatrick et al. [42] to 99%, according to Yikilmaz et al. [45]. Reporting reduced or absent intestinal wall enhancement is mandatory because it is a recognized predictor of transmural necrosis [40].

#### 3.1.2. Bowel Dilatation

Another essential and underestimated feature is intestinal dilatation, with a cut-off of 25 mm. Intestinal dilatation indicates that the myenteric plexus of the intestinal wall is dying, and the intestine, therefore, loses its sensitivity to contraction. It is thus a sign of advanced intestinal ischemia. Three independent predictors of transmural necrosis have been identified by Nuzzo et al. [15]: organ failure, serum lactate levels ≥ 2 mmol/L, and small bowel dilation. The risk of transmural necrosis increases with the presence of these factors, reaching 100% when all three are observed. Currently, dilatation is the factor most strongly correlated with intestinal necrosis and is the sign least subject to variability in assessment among readers [40]. However, this sign can only be considered indicative of intestinal necrosis if the diagnosis of AMI is certain; otherwise, isolated intestinal dilatation is non-specific [12].

#### 3.1.3. Pneumatosis

**Figure 4:**
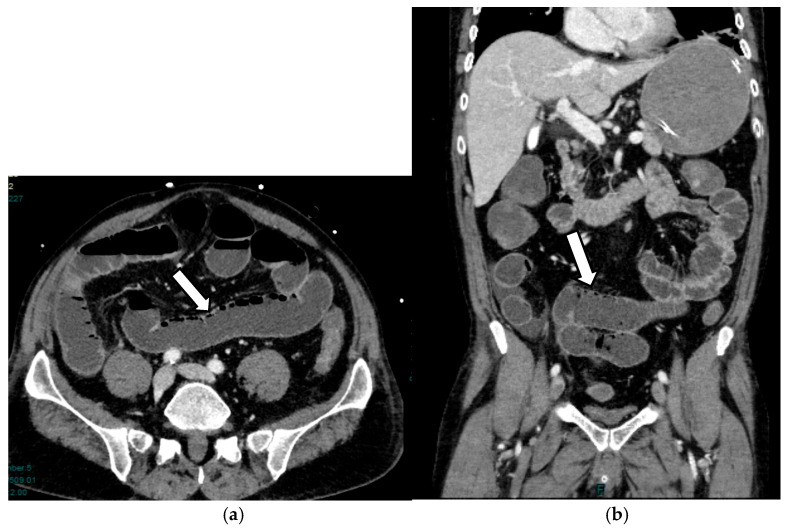
AAMI in a 56-year-old man: axial (**a**) and coronal (**b**) post-contrast CT images show dilated bowel loops, thinned bowel walls with decreased enhancement, and initial signs of pneumatosis (arrows).

In the late phase, wall necrosis becomes transmural: the typical radiological finding identifying this phase is pneumatosis of the intestinal wall, which may or may not be associated with gas in the portal system [40,46]. However, according to some studies, the interpretation of this sign is controversial, and the clinical significance of pneumatosis as a solitary radiological finding remains uncertain. Nuzzo et al. [15] estimate that only 5% of patients with transmural necrosis present with wall pneumatosis at CT; other studies show that patients with wall pneumatosis showed viable intestines following surgery [47,48]. In another large multicenter retrospective study, 60% of patients were found to have benign conditions [49]. Isolated pneumatosis without gas in the portal venous system is estimated to be associated with reversible lesions in more than 40% of patients (Figure 4).

**Figure 5:**
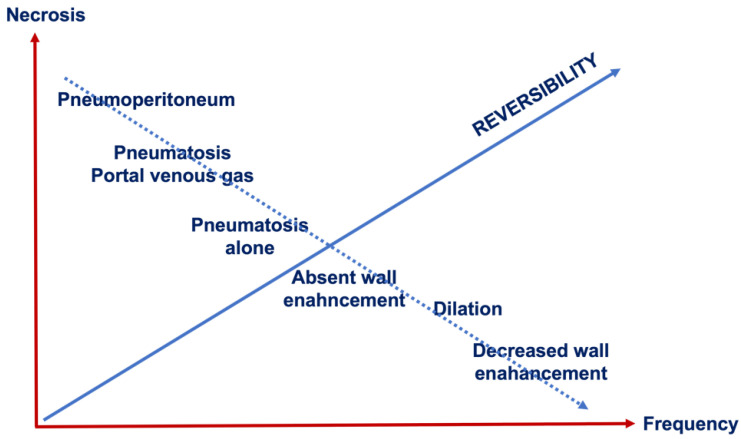
Different features of AMI (explanation in text).

Figure 5 shows the different features of AMI in a graphic: from left to right, we have the frequency, and from bottom to top, we have the probability of necrosis. Pneumoperitoneum almost certainly identifies transmural necrosis; wall pneumatosis, especially when associated with air in the portal venous system, determines a 90% probability of necrosis, pneumatosis alone may be associated with a 40% probability of viable bowel walls. Absent wall enhancement, dilatation of the bowel, and decreased wall enhancement are associated with necrosis in a lower percentage of cases. According to the most recent studies [50], the treatment strategy in patients diagnosed with acute mesenteric ischemia is based on intestinal viability: a combination of medical treatment with revascularization of the intestinal loops that are still viable (endovascular treatment or open surgery) and surgical resection of non-viable/necrotic intestinal loops is used. From left to right, we see a decrease in the possibility of necrosis, which correlates with an increase in the probability of reversibility of the lesions and a greater chance of therapeutic success without bowel resection. If AMI is treated in time with revascularization or bowel resection, the prognosis improves greatly, resulting in a 10–20% mortality rate in some cohorts [15,33]; hence, the interval between the onset of symptoms and the treatment is crucial.

## 4. Acute Venous Mesenteric Infarction (AVMI)

Venous mesenteric ischemia is a rare cause of AMI, accounting for approximately 5–15% of all occlusive ischemic cases. It is more frequently observed in females and typically affects younger individuals compared to those with acute arterial or non-occlusive mesenteric ischemia [51,52].

Venous occlusion is generally thrombotic, involving the mesenteric and portal veins, with etiologies like deep vein thrombosis in the lower limbs. These include conditions leading to venous stasis, hypercoagulability, and endothelial injury; this is collectively known as Virchow’s triad, which applies to all venous systems [53].

### 4.1. Risk Factor for AMVI

Key risk factors for AVMI include coagulation disorders, which can be categorized as primary (protein C, protein S, and antithrombin III deficiencies: plasminogen activator deficiency) and secondary (malignancies, myeloproliferative disorders, chronic inflammation, and cirrhosis).

Overall, hypercoagulable states and conditions causing reduced blood flow (more common in females), such as portal hypertension, diabetes mellitus, malignancies, and hepatic cirrhosis, are pivotal contributors [54].

Not all patients with abdominal venous thrombosis develop AMI. However, those with cardiovascular risk factors, particularly diabetes mellitus, are at increased risk of intestinal resection.

### 4.2. Clinical Presentation and Laboratory Findings

Patients typically present with acute, severe abdominal pain, often necessitating opioid analgesia. Laboratory tests frequently reveal leukocytosis, elevated C-reactive protein (CRP), increased lactate levels, and creatinine [51,52].

### 4.3. Diagnostic Imaging

Contrast-enhanced computed tomography (CT) is the diagnostic gold standard due to its comprehensive, rapid, and readily available nature. It enables direct visualization of the thrombus and provides a detailed assessment of the extent of bowel wall damage and associated complications [55,56,57].

- Pre-contrast phase: Recent thrombi may appear hyperdense, aiding early diagnosis.
- Arterial and venous phases: These highlight bowel wall thickening and layered enhancement, a hallmark of impaired venous outflow. This results in vascular congestion of the bowel wall and mesentery. Prolonged obstruction may lead to free abdominal fluid.

**Figure 6:**
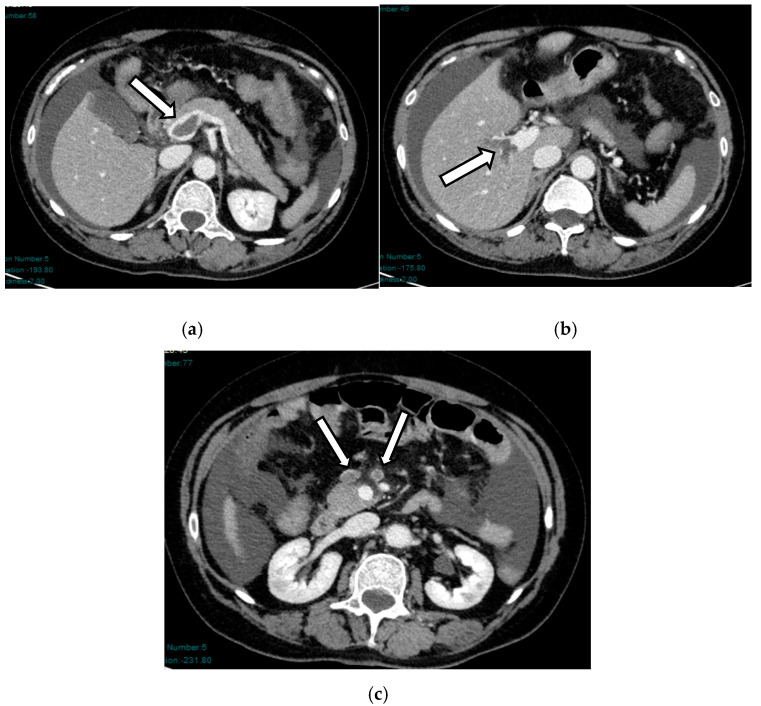
Thrombotic AMVI in a 64-year-old woman: axial post-contrast CT scans show large intraluminal filling defects (pointed out by arrows) in the portal vein (markedly dilated) (**a**), in the intrahepatic portal branches (**b**), and in the SMV and inferior mesenteric vein (IMV) (**c**).

**Figure 7:**
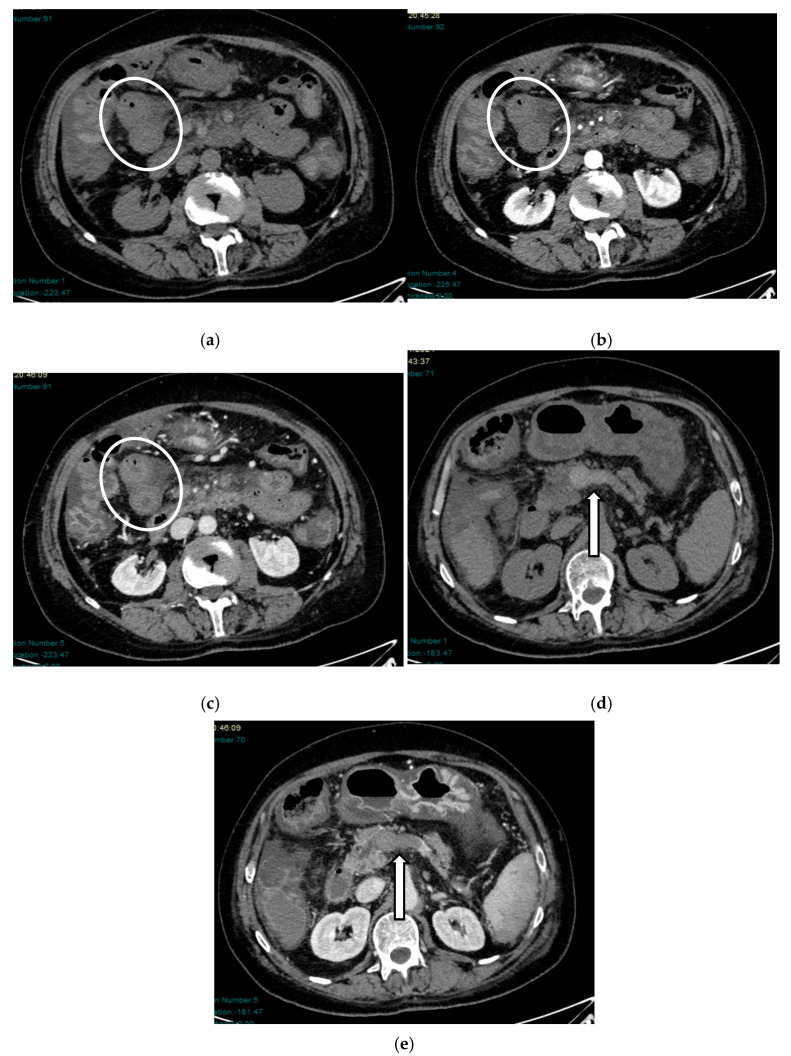
Thrombotic AMVI in a 60-year-old man: axial pre- and post-contrast scans show some jejunal loops with thickened and shaded walls and low enhancement (circles) (**a**–**c**). A marked mesenteric thickening caused by hemorrhagic infarction can also be appreciated. Extensive thrombosis of the portal vein appears hyperdense in an unenhanced CT scan (arrows) (**d**,**e**).

In the venous phase, the bowel walls often exhibit thickening with a multilayered appearance and reduced enhancement (“target sign”) [58]. The stratified appearance of the intestinal wall depends on the extent of intramural edema, ischemic congestion, and wall infarction, if the underlying cause is not solved [59]. Venous thrombi in the portal or superior mesenteric veins are commonly visible, often appearing spontaneously hyperdense on pre-contrast scans—a direct indicator of acute thrombosis (Figure 6 and Figure 7).

### 4.4. Pathophysiological Progression

Thickened, edematous bowel walls undergo increased permeability, initially leading to fat stranding in the perivisceral adipose tissue and subsequently to ascites. Prolonged ischemia results in bowel wall necrosis, which may manifest as pneumatosis intestinalis, an indicator of irreversible damage. If untreated, bacterial translocation and subsequent sepsis can occur.

**Figure 8:**
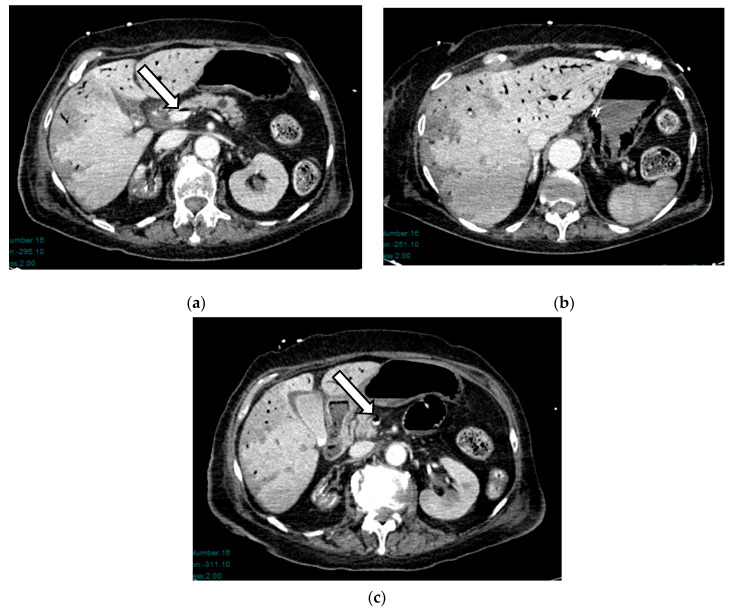
AMVI in an 84-year-old woman: axial contrast-enhanced CT scans show venous gas in the portal vein (arrow) and intrahepatic portal branches (**a**,**b**), as well as in the SMV (arrow) (**c**).

**Figure 9:**
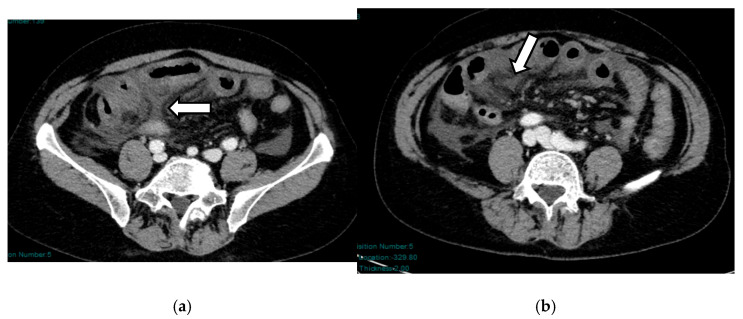
AMVI in a 64-year-old woman: axial contrast-enhanced CT scans show markedly thickened bowel walls of most of the ileal loops in the right iliac fossa and in the meso-hypogastric region, with reduced wall enhancing (**a**,**b**). Diffuse mesenteric thickening with free endoabdominal fluid (arrow).

Necrosis progresses more rapidly in venous ischemia (around 6 h) than in arterial ischemia (around 8 h) due to increased intraluminal content favoring bacterial proliferation. Gas produced within necrotic loops may enter the systemic circulation, resulting in portal pneumatosis (distinct from aerobilia) [48,60] (Figure 8 and Figure 9).

### 4.5. Management and Treatment of AVMI

Bowel wall thickening with a target appearance can develop within approximately 3 h, and beyond this window, surgical intervention with intestinal resection may be necessary [61]. Early intervention, before significant wall damage, allows for conservative management aimed at thrombus removal and correction of the coagulation imbalance [62].

Timely treatment is critical since AVMI is a potentially reversible condition. Early intervention can prevent necrosis or limit the extent of the affected bowel, with the duration of ischemia directly correlating with the length of bowel preserved [62].

## 5. Non-Occlusive Mesenteric Ischemia (NOMI) 

Non-occlusive mesenteric ischemia (NOMI) is due to vascular insufficiency due to low-flow states, hypotension (i.e., cardiogenic, septic, or hemorrhagic shock), or hypoxemia. Additional triggers can involve blunt abdominal trauma or major surgeries, such as cardiac or abdominal procedures. During hypo-afflux, the intestine suffers; the jejunal loops and the ascending colon are believed to be most influenced by hypoperfusion shock. Moreover, to the generalized state of hypoperfusion, parenchymatous organs such as the spleen or kidneys may develop ischemic foci [63]. The prevalence of NOMI varies widely depending on the studied population, with rates ranging from 6% [28] to 47% [64] among AMI cases. Typically, patients over 50 are more frequently affected by this condition. Among the types of AMIs, NOMI poses the greatest threat, with mortality rates reaching 58–70% due to its nonspecific clinical presentation and often subtle observations on contrast-enhanced CT (CECT) scans [65].

### 5.1. Vascular Involvement in NOMI

Key vascular observations on CECT include a reduced diameter of the superior mesenteric vein (SMV) and flattening of the inferior vena cava (IVC) [66]. While the SMA remains patent in NOMI, its diameter may appear diminished compared to prior imaging studies, with an average reduction of approximately 2 mm noted in some studies [67,68]. Poor intestinal arcades and intramural vessel visualization may also be observed [69].

### 5.2. Bowel Involvement in NOMI

#### 5.2.1. Ischemic Injury—Early Stage

In contrast to arterial occlusive acute mesenteric ischemia (AAMI), NOMI does not have a time-dependent progression. Instead, ischemia, reperfusion, and necrosis often coexist in a complex interplay [12]. CECT observations indicative of early ischemic damage include alternating regions of bowel wall thickening (from reperfusion injury) and thinning. Wall enhancement abnormalities, either increased or decreased, can also be present, often in a patchy distribution with normal intervening bowel segments [65]. The “target sign,” characterized by hypoenhancing submucosa between hyper-enhancing mucosa and muscularis propria or serosa, is not exclusive to NOMI but can also occur in arterial occlusions with reperfusion, venous ischemia, or even non-ischemic conditions like Crohn’s disease [58,66]. Bowel dilation is reported in 40% of cases, being more common in arterial occlusions compared to NOMI or venous occlusions [70]. Pneumatosis intestinalis, with or without portal venous gas, is identified in 30–40% of initial CECT scans [17]. Fat stranding often reflects an ongoing reperfusion process in NOMI [27].

In the early stage, the first approach to NOMI is focused on the underlying clinical condition: stopping pharmacological agents that contribute to the low-flow state may be the first approach to attempt an effective and valid intestinal reperfusion. Restoring circulatory volume and administering vasodilatory agents (i.e., prostaglandin) directly into the SMA can significantly enhance mesenteric perfusion [71]. Alternative infusible medications include papaverine and nitroglycerin.

#### 5.2.2. Necrotic Injury—Late Stage

**Figure 10:**
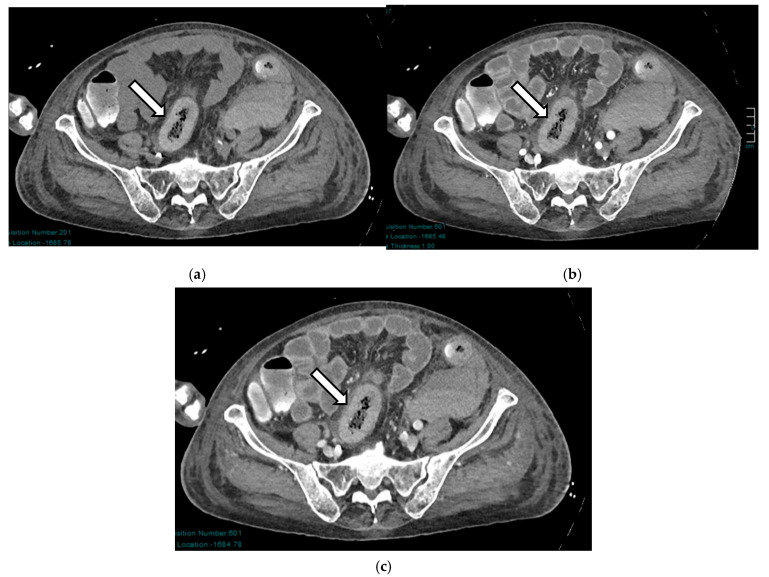
NOMI in a 78-year-old man: on axial pre- and post-contrast CT images, arrows show thickened bowel wall with mucosal hyperenhancing at unenhanced scans (**a**); in post-contrast CT scans, bowel walls show no enhancing (**b**,**c**).

Bowel resection is required in 50–65% of NOMI cases due to suspected transmural necrosis [17]. A distinguishing feature of NOMI is discontinuous segmental necrosis, which differs from the continuous necrosis observed in thrombus-associated mesenteric ischemia, where both the mesentery and intestine necrotize in the vascular territory [27]. Surgical intervention eliminates septic foci and prevents bacterial translocation associated with systemic necrosis. However, extensive resections carry the risk of short bowel syndrome or the need for permanent parenteral nutrition [40]. Hence, radiologists must be adept at identifying CECT features of AMI, especially NOMI, as well as signs predictive of necrosis (Figure 10).

## 6. Chronic Mesenteric Ischemia (CMI)

Chronic mesenteric ischemia (CMI), also known as “intestinal angina,” is a rare condition characterized by the chronic stenosis or occlusion of the celiac trunk, the SMA and/or the IMA, showing a higher prevalence in women (65–72% of cases) typically during their sixth decade of life and often associated with a significant smoking history [72,73].

### 6.1. Risk Factors for CMI

While atherosclerosis remains the primary etiology, rarer causes include vasculitis such as Takayasu, IgA vasculitis, or Buerger’s disease, as well as fibromuscular dysplasia, aortic coarctation, radiation enteritis, aortic or mesenteric dissections, retroperitoneal fibrosis, drug-induced damage, or post-surgical intimal hyperplasia [23,72,73].

A noteworthy, rare cause is Median Arcuate Ligament Syndrome (MALS), where the celiac trunk is compressed by the median arcuate ligament due to an abnormally high vascular origin, which is a condition that is typically relieved through surgical release of the ligament [2,74,75].

### 6.2. Diagnosis

Given that approximately 50% of these patients also suffer from peripheral or coronary artery disease, diagnosis requires a multidisciplinary approach combining clinical symptoms, such as postprandial abdominal pain, fear of eating, and weight loss, with radiological evidence [76].

CT is the gold standard for imaging, offering a sensitivity of 88–96% and a specificity of 94–100%, with an optimal protocol involving pre-contrast scans to detect calcifications, followed by arterial and venous phases to highlight stenosis and/or occlusion of the celiac trunk, the SMA and/or the IMA, and the development of collateral vessels [76,77].

Regarding intestinal findings, in compensated CMI, the bowel wall typically appears normal, with no evidence of acute parietal edema. In contrast, in cases of acute-on-chronic mesenteric ischemia (AOCMI), we can observe signs indicative of acute exacerbation, such as bowel wall thickening, pneumatosis intestinalis, and the presence of free intraperitoneal fluid [78]. In cases where MALS is suspected, acquisitions during both inspiration and expiration are particularly useful to demonstrate the respiratory-related changes in the compressive effect of the ligament [79].

### 6.3. Management and Treatment of CMI

While medical treatment focuses on risk factor modification, addressing smoking, hypertension, diabetes, and hypercholesterolemia to prevent disease progression, definitive management relies on revascularization procedures [73,75,80].

Table 2 provides a final synoptic summary of the different forms of mesenteric ischemia.

## 7. Predictor Signs of Transmural Necrosis

Risk factors for irreversible transmural necrosis (ITN) have been proposed to guide patient stratification. Calame et al. identified four essential indicators: absent bowel enhancement, bowel wall thinning, plasma bicarbonate concentration ≤ 15 mmol/L, and prothrombin rate < 40% [81]. Similarly, Nuzzo et al. reported that ITN risk in AMI (2% of whom were NOMI) correlates with factors such as organ failure, serum lactate > 2 mmol/L, and bowel loop dilation on CECT. The latter point is particularly stressed: intestinal dilatation indicates that the myenteric plexus in the intestinal wall is compromised, and the intestine, therefore, loses its sensitivity to contraction, so it is a sign of rather advanced intestinal ischemia. Intestinal dilatation is currently the one most strongly related to intestinal necrosis, and it is also the sign least subject to variability in assessment between readers. The probability of ITN increases significantly with the number of associated risk factors, ranging from minimal risk with one factor to high risk with two or more. In this regard, it is also crucial to remember that normal lactate values do not a priori exclude the hypothesis of acute mesenteric ischemia (AMI). Therefore, to avoid missing the diagnosis, a CECT scan is always mandatory when AMI is suspected [15].

## 8. Imaging Limitations

Despite several studies demonstrating the high sensitivity and specificity of CT for the diagnosis of AMI, various limitations must be recognized in clinical practice.

First, there are currently no universal guidelines providing a standardized protocol for CT acquisition. Most CT scans for the acute abdomen are requested for elderly patients or patients with significant comorbidities, and, given the lack of specific clinical markers, scans are often acquired suboptimally without contrast, leading to diagnostic difficulties. In clinical practice, the absence of both arterial and venous phases reduces CT sensitivity, as the misinterpretation of intestinal findings is frequent due to the broad differential diagnosis [14,19,82].

Furthermore, the goal is to diagnose AMI before advanced ischemia develops, but in the early stages, CT findings are subtle and easily missed, with vascular signs generally being more detectable than bowel wall changes [22]. A study by Kärkkäinen et al. on 27 patients with AOCMI and 20 controls demonstrated that one-third of patients with AOCMI lacked “ischemia-specific” signs [83].

This difficulty is particularly evident in NOMI, which carries a poor prognosis due to absent or non-specific CT findings that often delay diagnosis, particularly in the early stages [27,65,84]. A single-center study validated the limitations of conventional imaging by evaluating 75 confirmed NOMI cases against 39 suspected but excluded cases; using surgical or post-mortem findings as the gold standard, the research demonstrated that, among 75 confirmed cases, 19 showed no suggestive radiological signs, including 10 patients who already presented with intestinal necrosis [85].

While specific training in abdominal imaging can increase diagnostic confidence [86], the determining factor remains clinical suspicion [87].

Several studies have shown that AMI is underdiagnosed if not clinically suspected; for instance, Lehtimäki et al. analysed 95 patients and found that characteristic findings were reported in 97% of cases when the clinical question mentioned AMI, but only in 81% when it was omitted [17]. Nonetheless, as evidenced by the study of Anglaret et al., adopting a customized CT protocol can neutralize the diagnostic disadvantages when AMI is not clinically suspected [18].

Ultimately, maintaining a high index of clinical suspicion supports radiologists in detecting AMI in the emergency setting.

## 9. Conclusions

AMI is a rare cause of acute abdomen characterized by high mortality, which requires a high level of radiological expertise. CECT is the first-line imaging method due to its wide availability, even in emergency settings, fast acquisition times, and wide spatial resolution. It should always include a non-contrast acquisition, an arterial phase, and a venous phase, as well as MPR.

The main difference that the radiologist can make is to allow early diagnosis before reaching intestinal necrosis, which can influence prognosis greatly. The aim of this review is therefore to improve the ability to predict the risk of intestinal necrosis by illustrating the main predictive factors to look for in these patients.

## Figures and Tables

**Table 1:** Predictors of ischemic bowel necrosis.

Predictors of Ischemic Bowel Necrosis	
Wall features	Thinning
	Thickening
	Increased unenhanced attenuation
	Decreased/absent enhancement
Intestinal lumen	Dilatation
	Feces sign
Mesentery	Fat stranding
	Ascites
Ischemic lesions of other organs	

**Table 2:** CT characteristics in different forms of mesenteric ischemia.

	AAMI	AVMI	NOMI	CMI
Vessels	Intraluminal filling defect in the SMA	Intraluminal filling defect in the SMV	Non intraluminal filling defects	Chronic stenosis or occlusion of the celiac trunk, the SMV or the IMA Prominent collateral vessels Vascular calcifications
Bowel wall	Early AAMI: Bowel wall thinning with low enhancement	Early AVMI: Bowel wall thickening and dilatation, mucosal hyperenhancement	Dilated bowel loops with segmental hypoenhancing	Compensated CMI: regular bowel wall AOCMI: Bowel wall thickening, bowel wall pneumatosis
	Advanced AAMI: Bowel dilatation with no enhancement, bowel wall pneumatosis	Advanced AVMI: Bowel wall pneumatosis, intravenous pneumatosis	After reperfusion: wall thickening with mucosal hypoenhancement	

## Data Availability

No new data were created or analyzed in this study.

## Abbreviations

The following abbreviations are used in this manuscript:

AAMI	Acute Arterial Mesenteric Ischemia
AMI	Acute Mesenteric Ischemia
AMVI	Acute Venous Mesenteric Ischemia
CECT	Contrast-Enhanced CT
CMI	Chronic Mesenteric Ischemia
CTA	Computed Tomography Angiography
DECT	Dual-Energy CT
I-FABP	Intestinal Fatty Acid Binding Protein
IMA	Inferior Mesenteric Artery
IMV	Inferior Mesenteric Vein
ITN	Irreversible Transmural Necrosis
IVC	Inferior Vena Cava
MPR	Multiplanar Reformation
NOMI	Non-Occlusive Mesenteric Ischemia
SMA	Superior Mesenteric Artery
SMV	Superior Mesenteric Vein
